# Heath’s Practical Anatomy—First American from the Second English Edition

**Published:** 1870-11

**Authors:** 


Heath's Practical Anatomy—first American from the second English edition, edited with additions by William W. Keen, M.D., Lecturer on Pathological Anatomy in the Jefferson Medical College; Lecturer on Anatomy and Operative Surgery in the Philadelphia School of Anatomy.

This book in reality supplies a want long felt for a real *dissector's manual.* We have many dissector's manuals, but none of them have been all that the student could ask, until this volume made its appearance. All of our dissector's manuals have either been so small, as to be of little benefit to the beginner, or so large and voluminous as to take his whole time to find what he wanted. But Heath's Practical Anatomy, is a real help to anyone at the dissecting table, whether he be an experienced anatomist or a beginner. It is full and concise, descriptions clear, and illustrated with fine woodcuts, all calculated to be of the greatest possible assistance to the student. Dr. Keen, the editor, knowing from several years of successful teaching, what the exact wants of the American student are, has considerably modified the text of the original English edition, so as to bring it in accord with the method pursued in American dissecting-rooms. He has also corrected all errors, typographical and otherwise, and has made a number of important additions. Among which are the latest researches on the action of muscles. The Supinator Rudii Longus, for instance, has long been described as a supinator and flexors of the forearm, but it has been shown, within a few years, to be a powerful flexors of the forearm, its high origin on the humerous giving it its power.

There are also practical observations on the action of the Interossei, Omohyoid, and the most recent investigations on the ligamentous action of muscles, and of the nervous supply to the arytenoid muscle. Dr. Keen has also added some practical remarks on hernia, triangles of the neck, and has given some general rules for the relation of parts and the structure of organs. The directions for the dissection of the eye, are the best to be found in any work on anatomy. The additions also include directions for the preservation of subjects for dissection, and for making anatomical preparations, useful knowledge, not only for the student in the dissecting-room, but also to the practitioner.

After a careful examination of this book, we can say, that it is, all considered, the best book for the use of students, and those engaged in dissecting, with which we are acquainted.

